# Supplementary material for: Elastically driven intermittent microscopic dynamics in soft solids
Source: Nat Commun. 2017 Jun 21;8:15846. doi: 10.1038/ncomms15846 (PMC5482056; doi:10.1038/ncomms15846)
Supplement: Supplementary Information [file ncomms15846-s1.pdf]

Type of file: PDF
Size of file: 0 KB
Title of file for HTML: Supplementary Information
Description: Supplementary Figures

Type of file: MP4
Size of file: 0 KB
Title of file for HTML: Supplementary Movie 1
Description: Animation showing the evolution of the particles displacement of the athermal network ($k_{\rm B}T/\epsilon = 0 $) over a time window that covers a few rupture events. The color code is the same used in Fig.4 of the manuscript: most of the time nothing happens except after the rupture of a bond, which produces a long-range displacement field.

Type of file: MP4
Size of file: 0 KB
Title of file for HTML: Supplementary Movie 2
Description: Animation showing the particles displacement over a time window that covers a few rupture events (same as in Suplementary movie1) at finite temperature $k_{\rm B}T/\epsilon=10^{-3}$. One can see that the particles move continuously but no significant change in the strain field after the bond breaking, because the consequences of the rupture are screened by the thermal fluctuations.

Type of file: PDF
Size of file: 0 KB
Title of file for HTML: Peer Review File
Description:

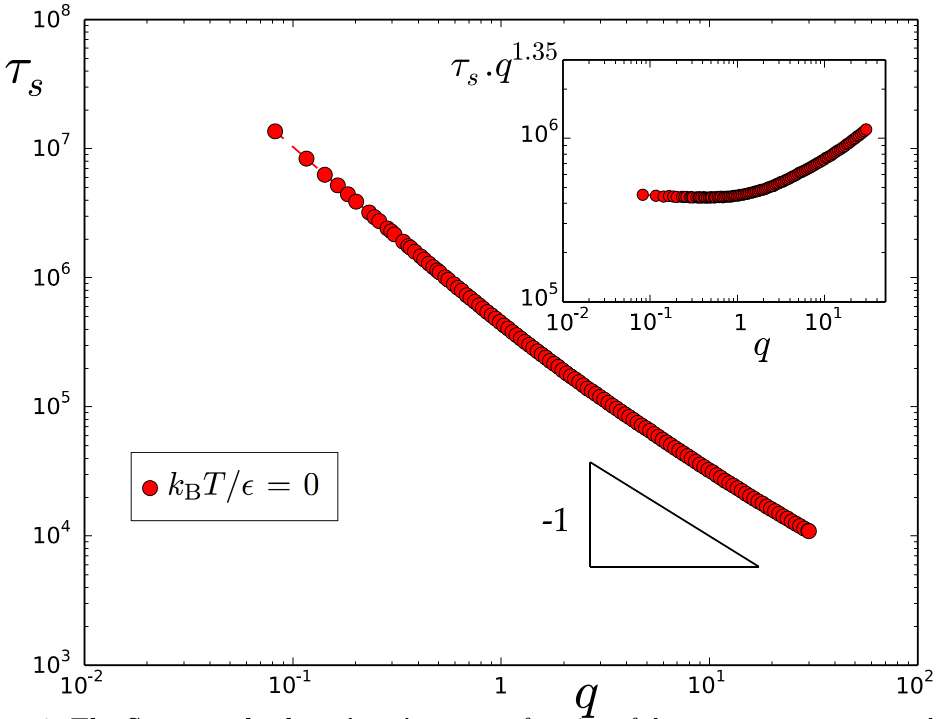

Supplementary Figure 1. **The Structural relaxation time**: τ_s as function of the wave vector q extracted from the incoherent scattering function, and for three different temperatures in the limit where thermal fluctuations are negligible, the inset shows the scaling for small wave vectors $\tau_s \sim q^{-1.35}$, the scaling at high q is ballistic $\tau_s \sim q^{-1}$ and the crossover between these scalings occurs at a length scale of the order of the mesh size.

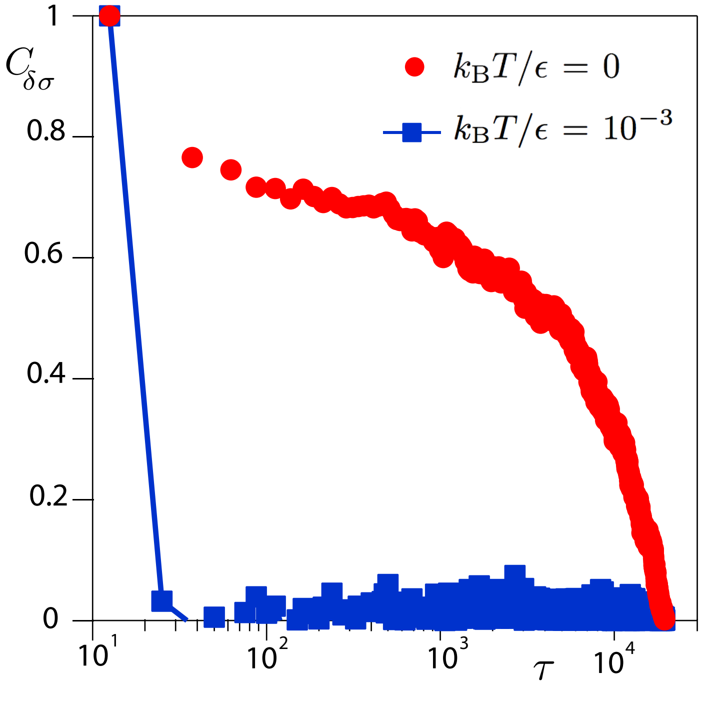

Supplementary Figure 2. **The stress fluctuations autocorrelation function**: $C_{\delta\sigma}$, where $\delta\sigma = \sigma_{xx} - \langle\sigma_{xx}\rangle$ measured over a time interval simulation corresponding to Fig.5a of the manuscript, involving 800 rupture events for the athermal regime for the athermal system (red) and when thermal fluctuations dominated $k_BT/\varepsilon = 10^{-3}$(blue).

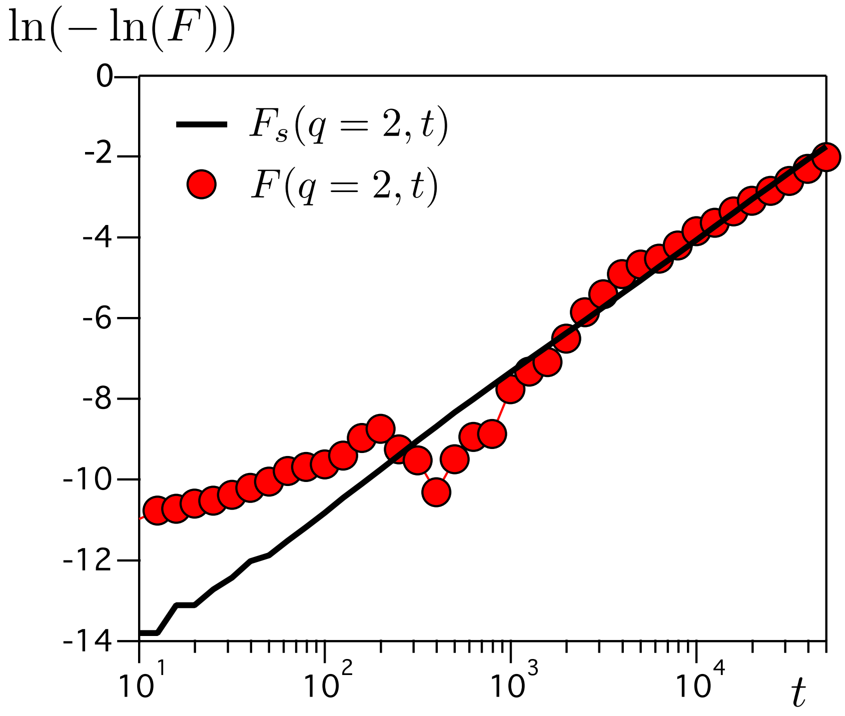

Supplementary Figure 3. **Coherent and incoherent scattering functions**: The decay of the coherent scattering function $F(q,t)$ and the incoherent $F_s(q,)$ for the same value of the wave vector $q=2$ showing that the β exponent in the last decay is the same.
